# Interaction of Cyclooxygenase-2 with *Helicobacter pylori* Induces Gastric Chronic Nonresolving Inflammation and the Formation of Syndrome of Internal Block of Static Blood in *Helicobacter pylori*-Related Gastric Diseases

**DOI:** 10.1155/2020/7340814

**Published:** 2020-04-06

**Authors:** Yun-kai Dai, Yun-zhan Zhang, Dan-yan Li, Xu Chen, Lin Gong, Qi Luo, Shao-yang Lan, Bin Chen, Jian-yu Wu, Zi-jing Zhang, Meng-xin Huang, Jin-tong Ye, Wei-jing Chen, Ru-liu Li, Ling Hu

**Affiliations:** ^1^Institute of Gastroenterology, Guangzhou University of Chinese Medicine, Guangzhou, Guangdong, China; ^2^First Affiliated Hospital of Guangzhou University of Chinese Medicine, Guangzhou, Guangdong, China

## Abstract

Cyclooxygenase-2 (COX-2) is an inducible enzyme stimulated by various inflammatory factors (IFs). Chronic gastritis is a classic model of “inflammation-cancer transformation” and *Helicobacter pylori*-related gastric diseases (HPGD) are specific ones of this model. Traditional Chinese Medicine (TCM) syndromes could play a predictive role in gastric histopathological evolution. To search for early warning evidence about “inflammation-cancer transformation,” this study is about to explore interaction of COX-2 with *H*e*licobacter pylori* (Hp) in HPGD with different TCM syndromes. All included subjects underwent endoscopy and biopsy. Hp infection was detected by rapid urease test and methylene blue staining. Histopathological characteristics and COX-2 expression in gastric mucosa (GM) were, respectively, observed by hematoxylin-eosin and Elivision™ plus. SPSS 18.0 and Stata 11.0 statistical software packages were used for statistical analysis. Results of immunohistochemical staining in this study showed COX-2 expression in Hp-positive patients was stronger than that in Hp-negative ones. Spearman' analysis indicated that degrees of both Hp infection and COX-2 expression were positively correlated with those of gastric inflammation and inflammatory activity. Compared with the relative normal group, both severe dysplasia group and gastric carcinoma group had more severe Hp infection and COX-2 expression. Compared with the nonsyndrome, syndrome of internal block of static blood (IBSB) had higher scores in semiquantitative analysis of COX-2 protein expression among TCM groups. Moreover, multivariate logistics regression analysis suggested that patients with Hp infection could increase the risk of IBSB. These results indicated that COX-2 interacting with Hp could play an important role in transforming gastric chronic nonresolving inflammation into carcinoma in subjects with HPGD, as well as inducing the formation of IBSB. HPGD together with IBSB could be an early warning evidence for GM with histopathological evolution from benign to malignant.

## 1. Introduction

Gastric carcinoma (GC) is the second dominant factor of mortality connected with malignant tumor. Accumulated evidence over the past several decades showed that GC is the final worst result of chronic gastritis (CG) [1, 2]. *Helicobacter pylori* (Hp), a spiral and Gram-negative microaerophilic bacterial pathogen, is epidemiologically associated with GC and has been categorized by the World Health Organization (WHO) as a group I carcinogen [3–5]. Because a major pathogenic factor of CG is Hp, these illnesses are named as Hp-related gastric diseases (HPGD) in the medical field. Usually, CG infected by Hp is a triggering event of gastric mucosal lesions, which contains a sequence of developmental stages as follows: chronic nonatrophic gastritis (CNAG), chronic atrophic gastritis (CAG), CAG accompanied by intestinal metaplasia (IM), accompanied by dysplasia (DYS), and even GC. The developmental stages are named as “inflammation-cancer transformation” model [6, 7]. And this classical model has become a hot research topic in terms of nonresolving inflammation-related cancer [8, 9].

Cyclooxygenase (COX) is a rate-limiting enzyme in transformation of arachidonic acid (AA) into prostaglandin (PG) [10]. Cyclooxygenase-1 (COX-1) and cyclooxygenase-2 (COX-2) are two different isoforms of this enzyme. Briefly, COX-1 is a constitutive enzyme mostly expressed in normal tissues and involved in producing PG to keep physiological functions work well. However, COX-2 is an inducible enzyme usually triggered by inflammatory factors (IFs). Several studies revealed that level of COX-2 protein, especially in existence of Hp infection, gradually increased with deterioration of gastric mucosal lesions, resulting in being highly expressed in early phase of precancerous gastric lesions [11–15]. Therefore, inducible COX-2 could be a key factor in early stage of GC.

With prevalence of traditional Chinese medicine (TCM) around the world, more and more patients put their concentrations on it. TCM syndromes, the same as a certain stage in gastric histopathological evolution, are comprehensive response to internal and external pathogenic factors in a certain stage of diseases. To a certain extent, a corporeity with gastric mucosal lesions accompanied by TCM syndromes can determine inherent tendency of this disease's development [9]. Our previous studies showed that Hp infection can activate abnormal expression of IFs in nuclear factor-*κ*B (NF-*κ*B) inflammatory signal pathway, thereby causing gastric mucosa (GM) with histopathological evolution from benign to malignant [9, 16–18]. Therefore, based on “inflammation-cancer transformation” model and syndrome differentiation (a TCM dynamic thinking), this study is about to explore interaction of COX-2 with Hp during the process of gastric inflammation-cancer transformation in HPGD, thereby providing practitioners with early warning evidence about gastric malignant pathology.

## 2. Methods

This research protocol was approved by Ethics Committee of the First Affiliated Hospital of Guangzhou University of Chinese Medicine. Furthermore, all included subjects volunteered to sign informed consent. The experiments conformed to the principles set out in the Declaration of Helsinki and the NIH Belmont Report.

### 2.1. Specimens

All volunteers were examined by endoscopy and diagnosed as CNAG, or CAG, or gastric ulcer (GU), or GC. Basic information and clinical data of all subjects were collected. And these corresponding specimens were blinded by digital number.

### 2.2. Criteria for Hp Infection

It was reference to Kyoto global consensus [19] and the second Asia-Pacific guidelines for Hp infection [20].

### 2.3. Criteria of Gastric Mucosal Histology

According to the updated Sydney system [21], histopathological changes in GM were detected by hematoxylin-eosin (HE) staining. Based on this, classification of HPGD was divided into six groups. Specifically, the relative normal group (NOR) referred to normal GM or only mild inflammation. Inflammation group (INF) referred to chronic inflammatory cell infiltration in GM. Atrophy group (ATR) referred to gastric gland atrophy without IM and DYS. Precancerous lesion group (PL) referred to gastric inflammation and gland atrophy with IM and nonsevere DYS. Severe DYS group (SD) referred to severe gland atrophy and inflammation accompanied by severe DYS. GC referred to different degrees of cancerization in GM.

### 2.4. Criteria of TCM Syndromes

It was reference to WHO international standard terminologies on traditional medicine in the western Pacific region [22] and syndrome differentiation in modern research of TCM [23]. TCM syndromes were differentiated by two professional practitioners. Specifically, nonsyndrome (NON) referred to dynamic equilibrium of body's metabolism. Syndrome of spleen-stomach dampness-heat (SSDH) was a pathological condition ascribed to accumulation of dampness-heat which impairs the functions of the spleen and stomach. Syndrome of liver qi invading the stomach (LQIS) was a pattern marked by dizziness hypochondriac pain, irritability, epigastric distension and pain, anorexia, belching, nausea, vomiting, and string-like pulse. Syndrome of internal block of static blood (IBSB) was a pathological product of blood stagnation, including extravasated blood and the blood circulating sluggishly or blood congested in a viscus, all of which may turn into pathogenic factor. Syndrome of spleen qi deficiency (SQD) was a pathological change characterized by qi deficiency with impaired transporting and transforming function of the spleen and stomach.

### 2.5. Inclusion

The inclusion criteria were as follows:

① Meeting the above diagnostic criteria, ② being eighteen years old and above, and ③ voluntary participation in this trial.

### 2.6. Exclusion

The inclusion criteria were as follows:

① Patients treated with proton pump inhibitors or Hp eradication therapy in the past month; ② patients with esophageal and gastric varices; ③ patients with other serious organ, blood, and nervous system diseases; and ④ pregnant and lactating women.

### 2.7. Materials and Chemical Regents

Both electronic endoscopy and binocular optical microscope were obtained from Olympus (Nagano, Japan; product model of electronic endoscopy: GIF-H290, GIF-Q260, and GID-XQ260; product model of binocular optical microscope: BX50F-3). Both tissue dehydrator and paraffin embedding machine were obtained from LEICA (Germany; product model of tissue dehydrator: TP1020; product model of paraffin embedding machine: EG1140H). A Rotary paraffin slicer was obtained from SHANDON (UK; product model: HANDON-AS325). A biological tissue sheet roasting machine was obtained from research institute of Yaguang medical electronic technology (Hubei, China; product model: YT-6). An electric constant temperature drying oven was obtained from Yuejin medical apparatus factory (Shanghai, China; product model: 101-1-BS). Polyclonal antibody of COX-2 was purchased from Abcam (Shanghai, China; batch no.GR287158). An Elivision plus kit was obtained from MAXIN (Fujian, China; batch no.1505074928). Rapid urease test (RUT) paper was purchased from Kedi Technology (Zhuhai, China; batch no.180102). A diaminobenzidine (DAB) color developing agent was purchased from Boster bioengineering co. LTD (Hubei, China; batch no.10109A32).

### 2.8. Source of Tissues

Two gastric mucosal tissues of each subject were used for this study. If there were lesions found under endoscopy, tissues would be taken from nidus (GU from its periphery). If not, tissues would be taken from greater and lesser curvature in gastric antrum.

### 2.9. Evaluation of Hp Infection and Histopathology

As for these collected gastric mucosal tissues, one was used for analyzing Hp status through RUT [24] immediately and the other was fixed in buffered formalin, embedded in paraffin and sectioned. Sections were stained with methylene blue (MB) [25] and HE, respectively. MB staining sections were applied to further evaluate Hp status. Either of the positive results of RUT and MB staining or both can be considered as Hp infection. In addition, HE staining sections were used for observing histopathological characteristics of each subject. After that, two specialists blindly and independently observed Hp infection and histopathology under binocular optical microscope. Subsequently, their results were scored as none, mild, moderate, and severe based on the updated Sydney system [21].

### 2.10. Immunohistochemical Evaluation

Immunohistochemical staining of COX-2 was conducted using Elivision plus kit (Fujian Maixin Biotechnology Development Co. Ltd., China). Briefly, sections were dewaxed and rehydrated by a graded alcohol series. After antigen retrieval conducted by heating at 100°C for 20 min in citrate buffer and blocking nonspecific blinding, sections were incubated with COX-2 polyclonal antibody (1 : 200; Abcam, Shanghai, China) at 4°C overnight. Then, these sections were incubated with corresponding moderate enzyme labeling secondary antibody for 40 min at room temperature. After DAB reaction, these sections were counterstained with hematoxylin and mounted with neutral resin. Under binocular optical microscope, intensity of immunostaining was scored by a semiquantitative method [26, 27]: briefly, 0 points for negative staining, 1 point for weak staining (light yellow), 2 points for moderate staining (brown), and 3 points for strong staining (dark brown). Besides, the percentage of these immunopositive cells was similarly scored: with 0 points, <5% of positive cells; 1 point for 5%∼25%; 2 points for 26%∼50%; 3 points for 51%∼75%; 4 points for over 75% positive cells. The average value of both scores of immunostaining intensity and immunopositive cells percentage was calculated as immunoreactivity scores. Based on this, COX-2 immunohistochemical staining was viewed as negative (−, <0.5), weak (+, 0.5–1.4), moderate (++, 1.5–2.5), and strong (+++, >2.5) positive.

### 2.11. Statistics

Statistical analysis was conducted using the SPSS 18.0 and Stata 11.0 statistical software package. All data were described as means ± standard deviation. Rank sum test or *χ*^2^-test was used for comparison among groups. Kruskal–Wallis *H* (*k*) test was used for independent samples among groups, and Mann-Whitney *U* test was used for pairwise comparison. One-way analysis of variance (ANOVA) was applied to test significance among groups. Multivariate logistics regression was analyzed to speculate risk factors. Spearman's analysis was used for correlations among degrees of Hp infection, inflammation, and COX-2 expression. ^*∗*^*P* < 0.05 was viewed statistically significant.

## 3. Results

### 3.1. Characteristics of Subjects

**Figure 1:**
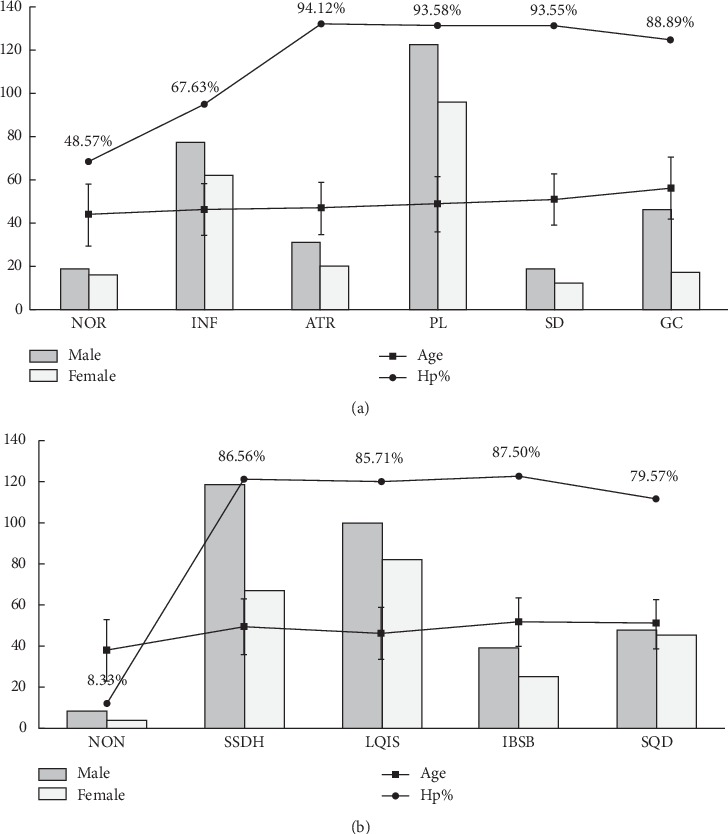
Characteristics of subjects. (a) Histopathological groups. Distribution of gender and age: *P* > 0.05. Hp% (*P* < 0.05): NOR versus other histopathological groups; SD versus GC. (b) TCM syndromes groups. Distribution of gender and age: *P* > 0.05. Hp% (*P* < 0.05): NON versus other TCM groups. Annotation: ages are presented as means ± standard deviation. Hp% = *H. pylori* positive rate. NOR = the relative normal group; INF = inflammation group; ATR = atrophy group; PL = precancerous lesion group; SD = severe dysplasia group; GC = gastric carcinoma group. NON = nonsyndrome; SSDH = syndrome of spleen-stomach dampness-heat; LQIS = syndrome of liver qi invading the stomach; IBSB = syndrome of internal block of static blood; SQD = syndrome of spleen qi deficiency.

A total of 537 subjects (314 males and 223 females) were recruited from September 2012 to December 2017 at digestive endoscopy center and gastrointestinal surgery in the first affiliated hospital of Guangzhou University of Chinese Medicine. As shown in Figure 1, distribution of gender and age had no statistical significance among histopathological and TCM groups. However, compared with NOR, other histopathological groups had significantly higher Hp infection rate, but this rate in GC was lower than that in SD (Figure 1(a)). In addition, compared with NON, other TCM groups had also significantly higher Hp infection rate (Figure 1(b)).

### 3.2. Situation of Hp Infection and Inflammation in GM

**Figure 2:**
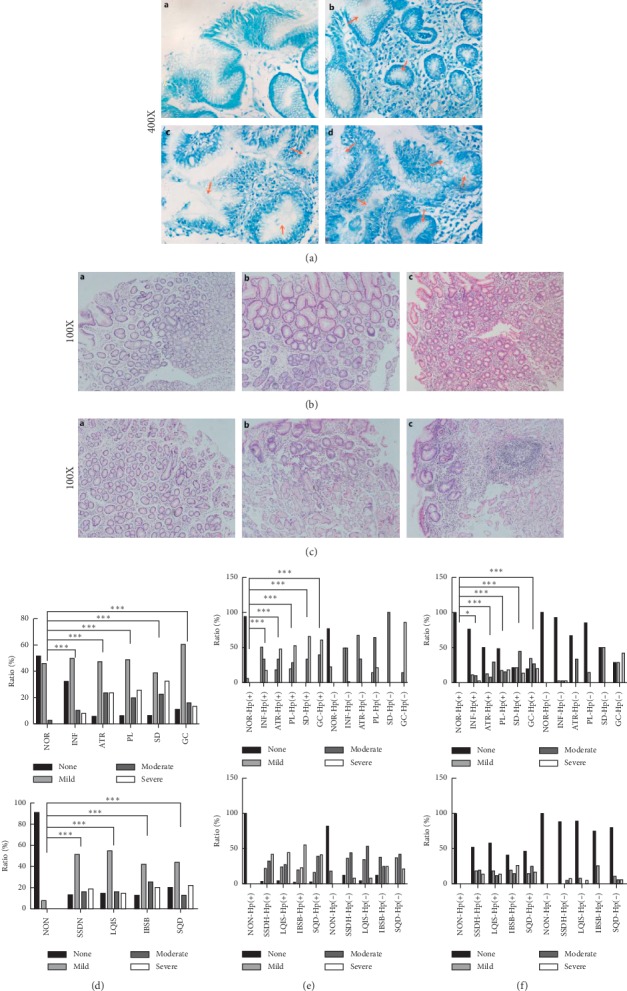
Situation of Hp infection and inflammation in gastric mucosa. (a) Situation of Hp infection. A–D: none, mild, moderate, and severe Hp infection. (b) Situation of inflammatory degree. A–C: mild, moderate, and severe inflammatory degree. (c) Situation of inflammatory activity. A–C: mild, moderate, and severe inflammatory activity. (d) Ratio of Hp infection. A: histopathological groups (*P* < 0.05); B: TCM syndromes groups (*P* < 0.05). (e) Ratio of inflammatory degree. A: histopathological groups (*P* < 0.05); B: TCM syndromes groups (*P* > 0.05). (f) Ratio of inflammatory activity. A: histopathological groups (*P* < 0.05); B: TCM syndromes groups (*P* > 0.05). Annotation: ^*∗*^*P* < 0.05; ^*∗∗*^*P* < 0.01; ^*∗∗∗*^*P* < 0.001.

After MB staining, Hp was clearly presented look like curved or rod-shaped in gastric epithelial surface and mucus layer. As shown in Figure 2(a), based on various amount of Hp dwelling, degree of Hp infection was considered as none, mild, moderate, and severe. In addition, gastric inflammatory degree was evaluated according to the density of inflammatory cells and depth of invasion. Evaluation of gastric inflammatory activity was based on the degree of inflammatory cell infiltration. These two were scored as mild, moderate, and severe (Figures 2(b) and 2(c)). As shown in Figures 2(d)(A)–2(f)(A) for Hp-positive, compared with NOR, SD had severer Hp infection and degrees of inflammation and inflammatory activity. As for Hp-negative, there was no significant statistical difference among histopathological groups. As shown in Figure 2(d)(B), compared with NON, other TCM groups had significant statistical differences in ratio of Hp infection and SQD had the highest ratio of severe Hp infection. However, degrees of inflammation and inflammatory activity had no significant statistical differences among these groups (Figures 2(e)(B)–2(f)(B)).

### 3.3. Expression of COX-2 in GM

**Figure 3:**
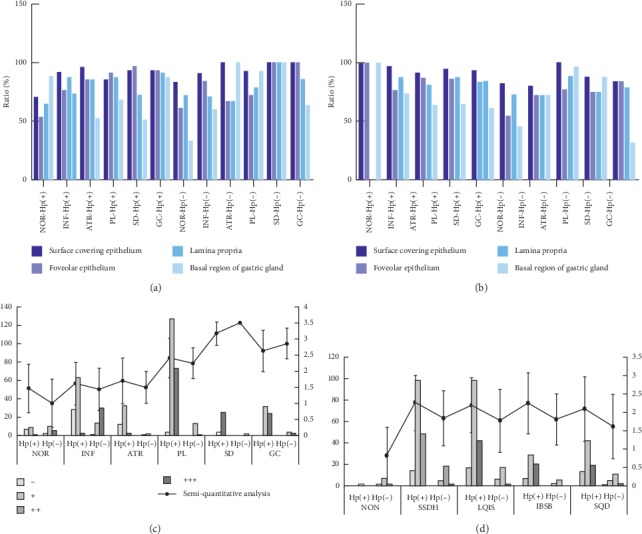
COX-2 expression in gastric mucosa. (a) Ratio of COX-2 expression in histopathological groups (*P* > 0.05). (b) Ratio of COX-2 expression in TCM syndromes groups (*P* > 0.05). (c) Results of semiquantitative analysis in histopathological groups (*P* < 0.05: Hp-positive vs. Hp-negative; NOR vs. SD, GC; SD vs. GC). (d) Results of semiquantitative analysis in TCM syndromes groups (*P* > 0.05). Annotation: semiquantitative analysis is presented as means ± standard deviation. Hp(+): *H. pylori* positive; Hp(−): *H. pylori* negative. −: negative expression of COX-2; +: mild expression of COX-2; ++: moderate expression of COX-2; +++: severe expression of COX-2.

**Figure 4:**
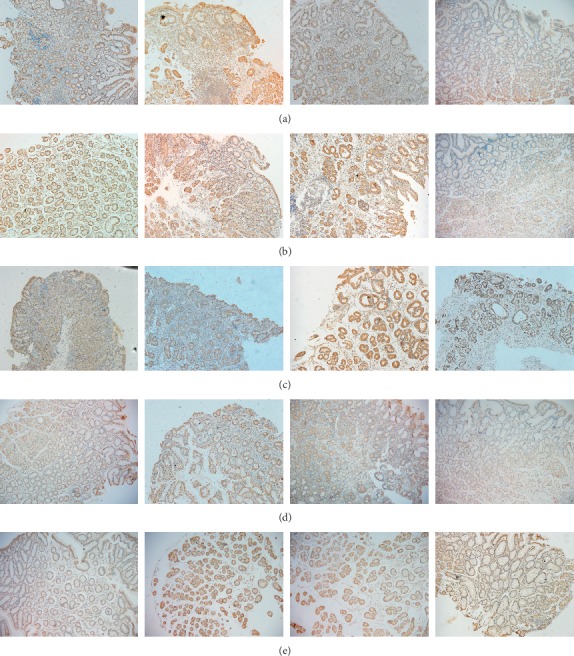
COX-2 protein expression of gastric mucosa in TCM groups (100x). (a–d**)** COX-2 expression in SSDH, LQIS, IBSB, and SQD. A–C: situation of COX-2 expression in different histopathological characteristics with Hp-positive; D: situation of COX-2 expression with Hp-negative. (e) COX-2 expression in NOR. A–D: situation of COX-2 expression in NON, SSDH, LQIS, and SQD.

The results of this study showed COX-2 expression, stained with yellow-brown color, was observed in gastric surface covering epithelium, foveolar epithelium, lamina propria, and basal region of gastric gland (Figures 3(a), 3(b), and 4(a)–4(e)). Moreover, whatever histopathological changes or TCM syndromes were, COX-2 expression was located in cytoplasm of gastric gland cells especially surface covering epithelium. Furthermore, COX-2 expression of Hp-positive tissues presented significantly stronger than that of Hp-negative ones (Figures 3(c) and 3(d)). Although SD and GC had more obvious COX-2 expression in comparison to NOR, degree of COX-2 expression in GC was inferior to that in SD. Besides, when GM was mildly inflamed or infected by Hp, COX-2 had weak expression (Figures 3(c) and 4(e)). As for TCM groups in Hp-positive, stronger COX-2 expression favored IBSB compared to other TCM groups (Figures 4(c) and 4(d)). However, in Hp-negative, COX-2 expression had no significant statistical difference (Figure 3(d)).

### 3.4. Multivariate Logistics Regression Analysis

In order to adjust influences (including gender, age, and Hp infection) on gastric histopathological evolution from benign to malignant, multivariate logistics regression analysis was performed. When NOR was used as a reference group, after adjusting gender and age, the results of Table 1 showed that patients with Hp-positive had higher risk of gastric malignant evolution than those with Hp-negative. Besides, after adjusting gender and Hp infection, risk of GC in HPGD patients over 55 years old was higher than those under 55. In addition, based on NON as a reference group, patients with Hp infection had higher risk of the onset of IBSB (RR = 72.048) among TCM groups (Table 2).

### 3.5. Spearman's Analysis

**Figure 5:**
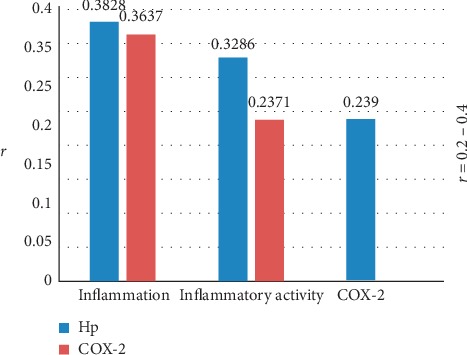
Spearman' analysis. Annotation: *r* correlation coefficient. *P* < 0.05: Hp infection degree versus COX-2 expression degree, degrees of inflammation and inflammatory activity; COX-2 expression degree versus degrees of inflammation and inflammatory activity.

In this study, spearman's analysis was conducted to observe correlations among degrees of Hp infection, COX-2 expression, inflammation, and inflammatory activity. The results of Figure 5 indicated degree of Hp infection was positively correlated with that of COX-2 expression, as well as degrees of inflammation and inflammatory activity. Moreover, degree of COX-2 expression was also positively correlated with degrees of inflammation and inflammatory activity. However, because the correlation coefficient (*r*) was from 0.2 to 0.4 in Figure 5, spearman's analysis among degrees of Hp infection, COX-2 expression, inflammation, and inflammatory activity indicated low correlations. Despite all of this, Hp infection had a little stronger correlation than COX-2 expression in degrees of both inflammation (*r*(Hp) = 0.3828 vs. *r*(COX-2) = 0.3673) and inflammatory activity (*r*(Hp) = 0.3286 vs. *r*(COX-2) = 0.2371).

## 4. Discussion

HPGD almost covers histopathological process of GM from mild to severe and benign to malignant. Previous studies have suggested that Hp infection is closely related to occurrence of GC [6–8]. Furthermore, gender also had an impact on clinical features of CG [28]. In this study, HPGD males outnumbered females, which may be associated with unhealthy living and dietary habits such as staying up, smoking, and excessive drinking. This result was consistent with the views that Kim et al. [29–31] believed that different genders' living and dietary habits were related to the onset of certain diseases. As for ages, the result of multivariate logistics regression analysis showed risk of GC in HPGD patients over 55 years old was higher than those under 55, suggesting that not only was age one of the risk factors on the onset of GC, but much attention should be paid to early GC screening among aged people.

Numerous studies showed that COX-2 protein is inactive under physiological condition, but it can be activated by various IFs [11–15]. As an environmental pathogenic factor, Hp infecting GM can induce IFs abnormal expression in NF-*κ*B inflammatory signaling pathway [9]. In this study, the results of immunohistochemical staining displayed COX-2 expression in Hp-positive patients were more obvious than those in Hp-negative ones. And the expression was mainly concentrated in gastric surface covering epithelium and foveolar epithelium. Moreover, the results of spearman' analysis showed that degrees of both Hp infection and COX-2 expression were positively correlated with those of inflammation and inflammatory activity. Compared with NOR, both SD and GC were severe Hp infection and COX-2 expression. Potential mechanisms of those phenomena were possibly associated with COX-2 overexpression triggered by IFs. Concretely speaking, Hp infection could greatly increase gastric acid secretion. Abundant hydrogen ion in gastric acid was easily to cause damage to gastric mucosal barrier and upregulated some IFs expression. Meanwhile, these IFs could make COX-2 overexpressed and its overexpression may affect proliferation and division of gastric epithelial cells, thereby aggravating mucosal lesions. With further deterioration in this condition, continuous Hp infection was unable to recover from a resolving inflammatory balance of anti-infection and anti-injury, which eventually led to irreversible injury in GM. These potential mechanisms coincided with the classical model of “inflammation-cancer transformation” in CG [6–9]. Therefore, we speculated interaction of COX-2 with Hp could play an important role in transformation of chronic nonresolving inflammation into carcinoma in HPGD. However, after cancerization, degrees of Hp infection and COX-2 expression were somewhat weakened and risk coefficient of Hp infection was also declined, potentially suggesting that the interaction may not be core risk factors after gastric carcinogenesis. Besides, this study also showed most NOR volunteers had weak positive of COX-2, possibly being connected with mild Hp infection or mild inflammation which made their bodies in a resolving inflammatory balance of anti-infection and anti-injury.

At present, roles of Hp infection and COX-2 expression have been extensively studied in the occurrence and development of HPGD especially precancerous gastric lesions [11–15, 32, 33]. Based on TCM dynamic thinking, this study investigated COX-2 expression for HPGD patients with TCM syndromes during gastric histopathological evolution from benign to malignant. As shown in Figure 3(d), IBSB had higher scores in semiquantitative analysis of COX-2 protein expression among TCM groups. And multivariate logistics regression analysis indicated that patients with Hp infection could increase the risk of IBSB (RR = 72.048). These findings suggested the formation of IBSB could be associated with Hp infection and COX-2 overexpression. On one hand, virulence factors released by Hp could make gastric epithelial cells degenerated and necrotic, causing inflammatory cell infiltrating into epithelial cells and thus triggering COX-2 overexpression. On the other hand, the overexpressed COX-2 could expand the existing inflammatory reaction and make it continuous, resulting in local microcirculation disturbance and blood stasis [34, 35]. Perhaps this was one of the reasons why IBSB (namely, syndrome of internal block of static blood) was closely related to Hp and COX-2. In addition, Figure 2(d)(B) also showed that the highest ratio of severe Hp infection was found in SQD, suggesting that gastric histopathological state of SQD was more vulnerable to Hp infection to some extent, mechanism of which probably derived from a histopathological change characterized by qi deficiency with impaired transporting and transforming function of the spleen and stomach, leading to impairment of gastric mucosal barrier function.

## 5. Conclusions

In summary, COX-2 interacting with Hp could induce gastric chronic nonresolving inflammation in subjects with HPGD. And the formation of IBSB could be closely related to Hp infection and COX-2 overexpression. Therefore, HPGD patients with IBSB could be an early warning signal for gastric malignant pathology and should be attracting enough attention clinically. In addition, SQD might be more vulnerable to Hp infection, to a certain extent, which provided potential clue for the formation and progress of HPGD and deserved further research.

## Figures and Tables

**Table 1:** Multivariate logistics regression analysis in pathological groups.

Histopathological groups	Independent variables	RR	SE	*z*	*P*
NOR	(Base outcome)				
INF	Hp	2.277^*∗*^	0.8830972	2.12	0.034
	Gender	1.020	0.3954924	0.05	0.959
	Age				
	<55	1.329	0.5989521	0.63	0.527
	<65	1.693	1.03691	0.86	0.390
	≧65	0.632	0.4200556	−0.69	0.490
ATR	Hp^*∗*^	17.324	11.90478	4.15	0.0001
	Gender	1.180	0.5480148	0.36	0.721
	Age				
	<55	1.131	0.6110357	0.23	0.820
	<65	1.585	1.111484	0.66	0.512
	≧65	0.566	0.4547304	−0.71	0.479
PL	Hp	15.422^*∗*^	6.801805	6.2	0.0001
	Gender	0.998	0.3875241	−0.01	0.995
	Age				
	<55	1.277	0.5846023	0.53	0.593
	<65	2.572	1.561198	1.56	0.120
	≧65	1.071	0.6752688	0.11	0.913
SD	Hp^*∗*^	15.125	12.26189	3.35	0.001
	Gender	1.226	0.638497	0.39	0.696
	Age				
	<55	1.393	0.8882281	0.52	0.604
	<65	4.059	2.973131	1.91	0.056
	≧65	0.988	0.8756455	−0.01	0.989
GC	Hp^*∗*^	6.890	3.706875	3.59	0.0001
	Gender	2.393	1.113171	1.88	0.061
	Age				
	<55	1.113	0.6630165	0.18	0.857
	<65^*∗*^	5.612	3.763102	2.57	0.010
	≧65^*∗*^	4.825	3.289365	2.31	0.021

^*∗*^ *P* < 0.05. RR: relative risk; SE: standard error. Hp: Hp (−) as a reference; Gender: female as a reference; Age: <45 as a reference.

**Table 2:** Multivariate logistics regression analysis in TCM groups.

TCM groups	Independent variables	RR	SE	*z*	*P*
NON	(Base outcome)				
SSDH	Hp	66.360^*∗*^	71.22943	3.91	0.0001
	Gender	0.797	0.5363312	−0.34	0.737
	Age				
	<55	1.103	0.7698166	0.14	0.889
	<65	2528084	2.19*E* + 09	0.02	0.986
	≧65	1.338	1.55997	0.25	0.803
LQIS	Hp	68.114^*∗*^	73.09308	3.93	0.0001
	Gender	0.532	0.3566124	−0.94	0.346
	Age				
	<55	1.014	0.7049037	0.02	0.984
	<65	1477533	1.28*E* + 09	0.02	0.987
	≧65	0.583	0.687404	−0.46	0.647
IBSB	Hp	72.048^*∗*^	80.58746	3.82	0.0001
	Gender	0.714	0.5035651	−0.48	0.633
	Age				
	<55	1.504	1.123849	0.55	0.585
	<65	3083635	2.67*E* + 09	0.02	0.986
	≧65	1.673	2.022528	0.43	0.670
SQD	Hp	41.117^*∗*^	44.61288	3.43	0.001
	Gender	0.507	0.3472169	−0.99	0.321
	Age				
	<55	1.871	1.344472	0.87	0.384
	<65	3893661	3.37*E* + 09	0.02	0.986
	≧65	1.892	2.242295	0.54	0.591

^*∗*^ *P* < 0.05. RR: relative risk; SE: standard error. Hp: Hp (−) as a reference; Gender: female as a reference; Age: <45 as a reference.

## Data Availability

Data used to support the findings of this study are included within the article.
